# Therapeutic potential of adipose derived stromal cells for major skin inflammatory diseases

**DOI:** 10.3389/fmed.2024.1298229

**Published:** 2024-02-23

**Authors:** Marina Ramírez Galera, Jesper Svalgaard, Anders Woetmann

**Affiliations:** ^1^The LEO Foundation Skin Immunology Research Center, Department of Immunology and Microbiology, Faculty of Health and Medical Sciences, University of Copenhagen, Copenhagen, Denmark; ^2^StemMedical A/S, Copenhagen, Denmark

**Keywords:** stem cells, ADMSCs, skin inflammation, therapy, cytokines

## Abstract

Inflammatory skin diseases like psoriasis and atopic dermatitis are chronic inflammatory skin conditions continuously under investigation due to increased prevalence and lack of cure. Moreover, long-term treatments available are often associated with adverse effects and drug resistance. Consequently, there is a clear unmet need for new therapeutic approaches. One promising and cutting-edge treatment option is the use of adipose-derived mesenchymal stromal cells (AD-MSCs) due to its immunomodulatory and anti-inflammatory properties. Therefore, this mini review aims to highlight why adipose-derived mesenchymal stromal cells are a potential new treatment for these diseases by summarizing the pre-clinical and clinical studies investigated up to date and addressing current limitations and unresolved clinical questions from a dermatological and immunomodulatory point of view.

## 1 Introduction

Inflammatory skin diseases are caused by a complex and self-sustaining interplay between multiple immune cell populations and skin cells via the release of pro-inflammatory cytokines, and are associated with impaired quality of life, social stigmatization and high incidence of severe comorbidities (1–3). Present therapies provide temporary amelioration of disease symptoms but offer no cure. Moreover, long-term treatment is associated with adverse effects and drug resistance. Thus, new therapeutic approaches are required to improve the life of patients with skin inflammatory diseases. At the forefront is adipose-derived mesenchymal stromal cells (AD-MSCs)-based therapy, which provides clinical promise due to its potent immunomodulatory and anti-inflammatory potential.

Since 1976, when Friedenstein et al. (4) discovered clonogenic progenitors from mouse bone marrow that would later be named mesenchymal stem cells by Caplan (5), there have been major efforts in investigating the use of these cells as a novel therapeutic agent for numerous incurable diseases. Mesenchymal stem cells are multipotent stromal-derived non-hematopoietic progenitor cells that reside in, and can be isolated from, various tissues of adult and neonatal origin. Among them, adipose tissue, bone marrow and cord blood derived cells are frequently used in human clinical trials. In order to standardize, validate and strengthen the increasing amount of preclinical and clinical data, the Committee of the International Society for Cellular Therapy (ISCT) established the minimal criteria characterizing human mesenchymal stem cells (6), and later on, proposed guidelines for its immunological characterization for clinical use (7, 8). Most recently, ISCT has published a position statement on nomenclature concluding that mesenchymal stem cells should be named mesenchymal stromal cells instead of stem cells (9). In this review MSCs stands for mesenchymal stromal cells.

AD-MSCs are a superior mesenchymal stromal cell source compared to bone marrow derived stromal cells (BM-MSCs) for several reasons. AD-MSCs are harvested from liposuction surgeries which are less painful and provide a higher percentage of progenitor cells compared to harvesting BM-MSCs (10). Briefly, discarded human subcutaneous adipose tissue (lipoaspirate) is enzymatically digested, and after several washes and centrifugation steps, the remaining pellet containing the stromal vascular fraction (SVF) is resuspended in appropriate media for subsequent treatment application or cell expansion. Of note, SVF is a combination of heterogeneous cell populations like lymphocytes, macrophages, smooth muscle cells, endothelial cells and its precursors, pericytes, pre-adipocytes and AD-MSCs. After isolation, *in vitro* culture of the SVF allows expansion and enrichment of adherent AD-MSCs. Expanded AD-MSCs are phenotypically characterized by flow cytometry and the ability to differentiate into osteogenic, chondrogenic and adipocyte cell lineages (11). It has been demonstrated that AD-MSCs have higher immunosuppressive capacity and lower expression level of major histocompatibility complex (MHC) class I when compared with BM-MSCs (12, 13). Both AD-MSCs and umbilical cord blood derived mesenchymal stromal cells (UC-MSCs) have proven to be equally effective in several *in vitro* experiments, however, due to ethical reasons and easier access to adipose tissue, AD-MSCs are preferred (14–16).

**Figure 1:**
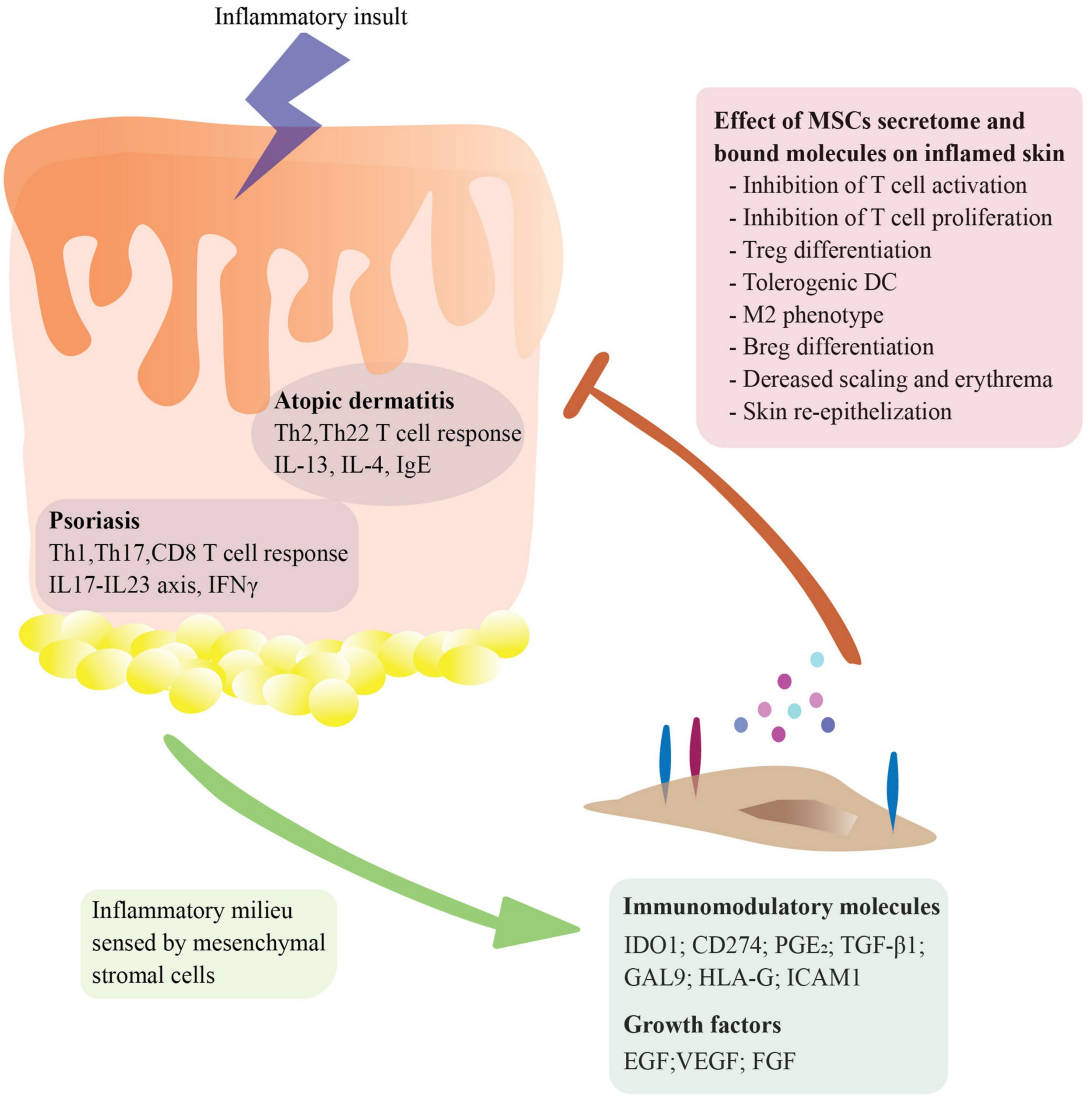
Molecular mechanisms underlying mesenchymal stromal cells (MSCs) therapeutic effect in major skin inflammatory conditions. MSCs sense the inflammatory microenvironment and respond to it by secreting a diverse array of molecules and expressing bound molecules in their cell surface, including homing receptors (CD44, ICAM1, CXCR4), cytokines (IL-6, CCL-2, CXCL8), growth factors (FGF, VEGF) and immunomodulatory molecules (CD274, IDO1, FasL). These mechanisms enable MSCs to regulate homeostasis, migrate to inflamed or damaged tissue sites, promote angiogenesis, facilitate tissue repair, and dampen ongoing inflammation. MSCs immunomodulation and immunosuppression affects different immune cell types including inhibition of dendritic cell differentiation, promotion of M2-like macrophage phenotype, suppression of mast cell activity, and modulation of T cell responses.

A defining characteristic of the immunopathogenesis of psoriasis and atopic dermatitis (AD) is an exacerbated T helper cell response. In psoriasis, the interplay between keratinocytes and different T cell subsets like Th1, Th17, CD8^+^ T and T regulatory cells (Treg) together with the presence of proinflammatory cytokines like IFN-γ, IL-22, TNF-α, IL-23, and IL-17 has been recognized as a key pathophysiological mechanism leading to a self-perpetuating inflammation and clinical manifestations (17). In atopic dermatitis, an aberrant type 2 immune response is caused by the interplay between fibroblasts, keratinocytes, and immune cells like Th2 and Th22 cells, type 2 innate lymphoid cells (ILC-2) and B cells. Which triggers the release of type 2 cytokines like IL-4, IL-5 and IL-13 as well as high levels of IgE (18). The potential therapeutic application of MSCs for skin inflammatory diseases arises from their contribution to skin re-epithelialization and their immunomodulatory capacity. As shown in Figure 1, MSCs have the ability to sense the inflammatory microenvironment and respond to it by secreting a wide range of molecules like homing receptors (CD44, ICAM1, CXCR4), cytokines (IL-6, CCL-2, CXCL8), growth factors (FGF, VEGF) and initiating cell–cell contact (CD274, FasL) (19, 20). These properties enable MSCs to maintain homeostasis, migrate to inflamed or damaged tissue sites and promote angiogenesis as well as tissue repair (21). MSCs contribute to skin re-epithelization by secreting many growth factors such as epidermal growth factor (EGF), transforming growth factor beta (TGF-β), vascular endothelial growth factor (VEGF), and basic fibroblast growth factor (bFGF) (22, 23). Moreover, MSCs immunomodulatory properties affect both the innate and adaptive immune system. Briefly, MSCs can inhibit the differentiation of monocytes into myeloid dendritic cells (24) and promote an M2-like phenotype (25, 26). In addition, MSCs secrete TGF-β1 and PGE_2,_ which inhibits the FceRI expression of mast cells, and therefore decreases IgE levels (27, 28). Also, they secrete immunosuppressive molecules like IDO-1 as well as immunosuppressive ligands like CD274 and HLA-G suppressing T cell proliferation while promoting differentiation into Treg cells (29–31). Furthermore, MSCs exert direct effects on B cells inhibiting their proliferation, plasma cell differentiation and chemotaxis (32, 33). Most importantly, accumulating data indicates that MSCs are not inherently immunosuppressive, but require stimulation by inflammatory mediators (i.e., IFN-γ) (34, 35). Lastly, MSCs are considered hypo-immunogenic cells because of their low expression levels of MHC class I and class II molecules. It was previously believed that MSCs were immune-privileged cells due to the lack of expression of MHC-II, but recent studies have demonstrated that upon inflammation (i.e., stimulation with IFN-γ) the expression of MHC-II molecules is upregulated (36–38). Nevertheless, MSCs lack co-stimulatory molecules like CD80 or CD86, which are required for full T cell activation (39), this enables MSCs to be used as an allogenic therapy with minimal risk for immune rejection. *In vitro* and *in vivo* studies showing the effects of AD-MSCs interaction with innate and adaptive immune cells are summarized in Supplementary Table S1.

## 2 Pre-clinical and clinical advances for atopic dermatitis and psoriasis

During the last years, there has been an increased use of AD-MSCs in clinical trials demonstrating not only the extensive treatment applicability of these cells but also their safety. Importantly, AD-MSCs have proven to be a safe and effective treatment of various experimental autoimmune and inflammatory skin disorders, including murine models of atopic dermatitis and allergic contact dermatitis (40–42). Intravenous injection of AD-MSCs reduced the serum IgE levels as well as the histological signatures of atopic dermatitis (41). In a similar study, intravenous injection of AD-MSCs resulted in a decreased number of degranulated mast cells, reduced IgE levels, and reduced concentrations of histamine, and prostaglandin E2 (PGE2) which resulted in the attenuation of atopic dermatitis clinical symptoms (42).

To date, there have been limited human case studies investigating the potential of MSCs as cell therapy for atopic dermatitis and psoriasis that indicate AD-MSCs potential safety and efficacy to treat these diseases. Autologous AD-MSCs were used in treatment of two patients with psoriasis vulgaris (PV) and psoriatic arthritis (PA) respectively. After two intravenous injection, the PA patient had a decrease in Psoriasis Area and Severity Index (PASI) from 21.6 to 9.0. The PV patient, who was previously dependent on methotrexate, experienced a decrease in PASI from 24.0 to 8.3 after three infusions and the effect was sustained for 9.7 months without methotrexate (43). In another case study, intravenous injection of the stromal vascular fraction (SVF) obtained after adipose tissue digestion, resulted in a significant decrease in symptoms with a noticeable difference in skin quality appearance (44). Psoriasis area and severity index score was reduced from 50.4 at baseline to 0.3 at 1 month follow-up. Furthermore, the patient did not report any safety concerns and did not experience any severe adverse events (44). Another study reported that AD-MSCs conditioned medium injected into a patient’s psoriasis plaque, resulted in complete abolishment of the severe psoriatic plaque within 1 month of treatment. The PSSI score reduced from 28 to zero, and regression of the disease continued for 6 months of follow-up. The patient did not take any other medication during the follow-up period of 6 months and did not experience any adverse side effects during the remainder of the study. The conditioned media consisted of the harvested supernatant of *in vitro* expanded AD-MSCs in serum-free DMEM. According to the authors the supernatant of AD-MSCs contains paracrine factors like growth factors, chemokines and cytokines, responsible for the healing and regenerative effect, however, they did not characterize the secretome further (45).

Currently, there are 11 ongoing phase I and/or phase II clinical studies using MSCs to treat psoriasis and 4 phase I clinical studies using MSCs to treat atopic dermatitis. Among these, a Phase I single group clinical study (NCT02888704) aims to evaluate the safety, tolerance, and efficacy of autologous AD-MSCs intravenous injection in patients with moderate to severe, subacute, and chronic atopic dermatitis. To date, no results are available, but in 2022, the same group completed a 1 year long-term observational follow-up study (NCT03252340) to evaluate safety of treated patients from the NCT02888704 trial. In the continuation of this study, researchers are recruiting more patients to start a phase II open label two group study to evaluate efficacy and safety for up to 5 years long-term observational study (NCT04137562).

Another research group is conducting several clinical trials using allogeneic AD-MSCs for patients with moderate to severe psoriasis. In 2017 they started a phase I and phase II study to evaluate safety and efficacy of intravenous infusion of 0.5million cells/kg of allogeneic AD-MSCs at week 0, 4 and 8 for a total of 12 weeks study (NTC03265613). They have also started other clinical studies with same time points to evaluate efficacy and safety of intravenous infusion of 2 million cells/kg of allogeneic AD-MSCs in combination with Calcipotriol Ointment (NCT03392311), Calcipotriol ointment plus PSORI-CM01 Granule (NCT04275024) as well as the use of allogeneic AD-MSCs in combination with PSORI-CM01 or Gu Ben Hua Yu formula (NCT04785027). Both PSORI-CM01 and Gu Ben Hua Yu are traditional Chinese medicinal herbs that are taken orally once a day for 12 weeks.

MSCs-derived exosomes have drawn significant interest for their immunomodulatory and regenerative capabilities (46). Exosomes are extracellular vesicles (EVs) containing proteins, lipids, RNAs, metabolites, growth factors, and cytokines that play a crucial role as essential regulators of intercellular communication in numerous physiological and pathological processes. Interestingly, a phase 1, open-label study aims to determine safety and tolerability of the topical application of MSCs-derived exosome ointment to treat psoriasis in healthy volunteers. Specifically, 100 μg of MSCs—exosomes/g ointment plus Vesiderm liposome cream 3 times per day during 20 days with a gap of 4 h between 3 doses (NCT05523011).

To date, there have been no results posted from all the above-mentioned ongoing clinical trials. Most recently, the results from a phase I clinical trial (IRCT20080728001031N24) investigating subcutaneous injection of allogeneic AD-MSCs in psoriatic patients have been published. After 6 months follow-up, data suggests that AD-MSCs treatment was safe and effective as no major severe effects were observed in any patient, and most importantly, the scaling, thickness and erythema of the lesions were decreased in all the of 5 patients at different levels of efficacy (47). A summary of the above-mentioned preclinical and clinical studies can be found in Table 1.

**Table 1:** Summary of finalised and ongoing studies investigating the use of AD-MSCs to treat atopic dermatitis and psoriasis, highlighting both preclinical findings in animal models and clinical findings in human subjects.

Disease	Type of study	Administration and number of cells	AD-MSCs source and cell passage	Outcome	Adverse effects	Reference
Allergic contact dermatitis (ACD)	Pre-clinical study using C57BL/6 mouse model	Intravenous injection (tail)—unknown cell number	Mouse allogeneic *in vitro* expanded until passage 4	Ear thickness was suppressed in mice injected with AD-MCs	Not mentioned	(40)
Atopic dermatitis (AD)	Pre-clinical study using NC/Nga mice model	Intravenous injection—2 × 10^5^or 2 × 10^6^ cells/200 μL normal saline	Human *in vitro* expanded (unknown passage)	Reduced gross and histological signatures of AD, as well as serum IgE level	No adverse effects observed	(41)
Atopic dermatitis (AD)	Pre-clinical study using BALB/c mouse model	Intravenous injection of 1 × 10^6^ cells in 100 μL phosphate buffered saline	Human *in vitro* expanded until passage 5	Attenuation of clinical symptoms associated with AD, decreased numbers of degranulated mast cells (MCs), IgE level, amount of histamine released, and prostaglandin E2 level	No adverse effects observed	(42)
Psoriasis	Case report including 2 patients with psoriasis vulgaris (PV) and psoriasis arthritis (PA) respectively	Intravenous injection of 0.5–3.1 × 10^6^ cells/kg in normal saline solution	Human autologous AD-MSCs *in vitro* expanded until passage 3	PA patient experienced a decrease in PASI from 21.6 to 9.0 and PV patient experienced a decrease in PASI from 24.0 to 8.3	No serious adverse events were noted for either patient	(43)
Psoriasis	Case report for 1 patient with severe psoriasis	Intravenous injection (unknown number of cells)	Human autologous AD-MSCs stromal vascular fraction (SVF)	Psoriasis plaques were completely resolved 1-month post single injection and continued to be resolved at 12 months follow up. Psoriasis area and severity index score went from 50.4 at baseline to 0.3 at 1 month follow-up	No adverse effects noted	(44)
Psoriasis	Case report including 1 patient suffering from psoriasis vulgaris	Topical application in psoriatic plaque once a day of 3 kDa concentrated media	Human allogeneic AD-MSCs *in vitro* passaged until P2	Complete abolishment of the severe psoriatic plaque within 1 month of treatment. The PSSI score reduced from 28 to zero, and regression of the disease continued for 6 months of follow-up	No adverse effects noted	(45)
Atopic dermatitis	Phase I single group clinical study (NCT02888704 & NCT03252340)	Intravenous injection of 1.0 × 10^8^ or 3.0 × 10^8^ cells	Human autologous AD-MSCs (unknown passage)	No results publish to date	No results publish to date	
Atopic dermatitis	Phase II randomized open label two group study (NCT04137562)	Intravenous injection of 0.5 × 10^8^ cells/5 mL	Human autologous AD-MSCs (unknown passage)	No results publish to date	No results publish to date	
Psoriasis	Phase I and phase II single group study (NTC03265613)	Intravenous infusion of 0.5 × 10^6^ cells/kg	Human allogeneic AD-MSCs (unknown passage)	No results publish to date	No results publish to date	
Psoriasis	Phase I and phase II single group study (NCT03392311; NCT04275024; NCT04785027)	Intravenous infusion of 2 × 10^6^ cells/kg	Human allogeneic AD-MSCs of unknown passage in combination with Calcipotriol ointment (NCT03392311), Calcipotriol ointment plus PSORI-CM01 Granule (NCT04275024), PSORI-CM01 formula or Gu Ben Hua Yu formula (NCT04785027)	No results publish to date	No results publish to date	
Psoriasis	Open-label phase I clinical trial (IRCT20080728001031N24)	Subcutaneous injection of 1 × 10^6^ or 3 × 10^6^ cells/cm^2^	Human allogeneic AD-MSCs (unknown passage)	Significant reduction of psoriatic plaque scaling, thickness, and erythema as well as decreased PASI scores	No adverse effects observed	(47)

## 3 Limitations and unresolved clinical questions

Despite the increased tendency to explore the clinical safety and efficacy of AD-MSCs for skin inflammatory diseases, there are a limited number of studies and most of them are case reports with few numbers of patients and no control groups. Results from ongoing clinical trials should provide data to demonstrate safety and efficacy. This review emphasizes the need to address both pre-clinical and clinical level gaps concerning culture methods and conditions of cell expansion (i.e., 2D vs. 3D), donor-specific differences (i.e., sex, age, body localization and donor’s health), as well as cell heterogeneity, which cell passage to use, ideal administration route, distribution of the cells after administration, cell dose and timing.

Current advancements using skin constructs are focused in the use of AD-MSCs regenerative properties for wound healing and skin re-epithelialization (48), however, for the treatment of skin inflammatory diseases, it is pivotal to develop novel mechanistic and functional assays to understand how AD-MSCs influence stromal cells like keratinocytes and fibroblasts, as well as the immune cells residing in the skin, including resident memory T cells, as these are considered important for local recurrence of skin inflammation in, e.g., psoriasis (17). A recent study, investigating the effects of AD-MSCs exosomes in a particulate matter (PM)-induced model of atopic dermatitis, found that PM-AD model treated with AD-MSCs exosomes had a decreased expression of proinflammatory cytokines like IL-6, IL-1β and IL-1α and a higher expression of skin barrier proteins like loricrin and filaggrin, compared to untreated PM-AD model (49).

As previously mentioned, MSCs acquire immunosuppressive capacity only in an inflammatory microenvironment. Most of the studies have focused on investigating immunomodulatory potential of IFN-γ primed MSCs, and most recently, others have focused on combination of IFN-γ and TNF-α demonstrating an increased immunosuppressive and immunomodulatory potential via membrane bound and secreted factors (i.e., IDO-1, ICAM-1 CD274, TGF-β, PGE2) (50, 51). However, from a therapeutical point of view, it would be highly interesting to understand how combinations of other cytokines known to drive skin inflammatory conditions (i.e., IL17A, IL23, IL-4/13, IL-33, TSLP etc.) affect MSCs immunosuppressive and immunomodulatory potential. And most importantly, how different inflammatory microenvironments affect MSCs cell heterogeneity.

Several studies propose using serum and xeno-free culture platforms for MSCs cell expansion (52), however, most of the experiments studying the effect of AD-MSCs *in vitro* cell expansion use foetal bovine serum (FBS) as a growth supplement for the cell culture. FBS is no longer the recommended supplement to culture ASCs for clinical purposes because of its potential immunogenicity and possible prion/zoonotic transmission. Instead, it has been demonstrated that pooled human platelet lysate (pHPL) is a better supplement because it enhances AD-MSCs viability and proliferation (53–55). Several *in vitro* studies have investigated the effect of long term culture in both cell senescence and immunomodulation, but differ notably from the optimal cell passage to use (56–58). Some studies indicate that the ideal cell passage for *in vitro* and *in vivo* experiments is passage 6 (56), others point that AD-MSCs do not alter their characteristics up to passage 10 (59) and others suggest using AD-MSCs at earlier passages than 5 (60). The difference in these results could indicate the importance of standardization of cell isolation and culture methods (e.g., seeding density, days in culture between passages etc.). Recent data indicates that expanding AD-MSCs in 3D cultures ameliorates senescence-related changes compared to two-dimensional (2D) cultures (61). However, the effect on AD-MSCs immunomodulatory and regenerative properties still requires clarification.

Studies have compared immunomodulatory and regenerative capacities of both allogeneic versus autologous MSCs, indicating that allogeneic MSCs efficacy is not inferior to autologous (62, 63). However, the impact of utilizing autologous or allogeneic MSCs is subject to the specific patient’s condition and comes with both positive and negative aspects. The comparative therapeutic advantages of employing autologous versus allogeneic MSCs lack definitive conclusions (64).

Equally important for AD-MSCs therapy success is donor specific-differences. Recently, a study has shown that AD-MSCs from female donors have a higher immunomodulatory and immunosuppressive capacity than male donors (65). It was also demonstrated that AD-MSCs from old donors have a lower cell viability and proliferation compared to younger AD-MSCs donors (66). In contrast, a new study characterizing the transcriptional and DNA methylation landscape of AD-MSCs from donors differing in age, concluded that cell passage has a greater effect on the stemness of ADSCs than donor age (67). Another aspect to take into consideration is the anatomical localization where fat tissue is taken from as well as donor’s health. A study comparing the inflammatory response of AD-MSCs derived from subcutaneous and visceral adipose tissue from obese, type 2 diabetic and healthy donors, showed that the unhealthy donor derived AD-MSCs had a lower immunomodulatory and immunosuppressive capacity compared to healthy (68). Moreover, a recent study demonstrated that AD-MSCs isolated from breast had superior immunomodulatory and antioxidative capabilities than AD-MSCs isolated from the abdomen (69).

Lastly, MSCs cell heterogeneity remains to be fully elucidated. To this end, techniques like single cell RNA-sequencing are enabling a better understanding and characterization of MSCs subpopulations. Some studies are already identifying gene sets for cell heterogeneity concluding that it can be due to ECM associated immune regulation, antigen processing and senescence, however this is based on the analysis of AD-MSCs, BM-MSCs and UC-MSCs together, lacking individual tissue origin subpopulation comparisons (70). Recent studies are demonstrating that within the MSC population there are several subpopulations in regard to stemness, functionality (immunomodulation) and cell proliferation. These studies contribute greatly to the knowledge about the use of MSCs for clinical translational purposes, however further studies need to be done to understand the application effect of such cells in the clinic (71, 72).

## 4 Conclusion

In conclusion, stem cell-based therapy for the treatment of inflammatory skin diseases remains an undeveloped research field with few studies published to date. Considering the available preclinical and clinical data, the immunomodulatory and immunosuppressive properties of AD-MSCs hold strong potential for its use as cell therapy for inflammatory skin diseases. Working towards a standardization of isolation and culture protocols for AD-MSCs *in vitro* expansion will be important for AD-MSCs therapeutic success in clinical application.

## Author contributions

MG: Conceptualization, Investigation, Writing – original draft. JS: Writing – review & editing. AW: Conceptualization, Funding acquisition, Supervision, Writing – review & editing.
