# Supplementary material for: Therapeutic potential of adipose derived stromal cells for major skin inflammatory diseases
Source: Front Med (Lausanne). 2024 Feb 23;11:1298229. doi: 10.3389/fmed.2024.1298229 (PMC10921940; doi:10.3389/fmed.2024.1298229)
Supplement: Supplementary file 1 [file Table_1.DOCX]

Supplementary Material

1. **Supplementary table 1**

| **Reference paper** | **Outcome** |
| --- | --- |
| Tavares T.R. et al(1) | IFN-γ licensed-AMSCs presented an elevated expression of indoleamine 2,3-dioxygenase (IDO), accompanied by increased ICAM-1, as well as a higher immunosuppressive potential, compared to unlicensed AMSCs. |
| Wang Y. et al(2) | ASCs suppressed DC maturation. In addition, ASC-treated mature DCs showed higher levels of TGF-β1, IL-10, and IDO expressions, as compared to that in matured DCs (mDCs) alone. ASC-treated mDCs co-cultured with CD4+ T cells revealed a significant higher percentage of Treg than mDC without treatment. |
| Gornostaeva A. et al (3) | Allogeneic ASCs do not enhance the activation of unstimulated immune cells and can provide supportive functions. |
| Zhang W. et al (4) | Transplantation of ADSCs could significantly inhibit autoimmune progression in MRL/lpr mice and the efficacy of ADSCs was comparable to that of CTX. |
| Wang Y. et al (5) | The ADSCs reduced the viability of Jurkat T cells and downregulated the transcription of tumour necrosis factor-α and transforming growth factor-β1. |
| Zhou K. et al (6) | Immunosuppression of human adipose-derived stem cells on T cell subsets via the reduction of NF-kappaB activation mediated by PD-L1/PD-1 and Gal-9/TIM-3 Pathways |
| Blazquez R. et al (7) | Exosomes derived from ASCs exerted an inhibitory effect in the differentiation and activation of T cells as well as a reduced T cell proliferation and IFN-γ release on in vitro stimulated cells. |
| Cho K. et al (8) | Extracellular vesicles from ASCs ameliorated Th2-mediated inflammation induced by Aspergillus protease antigen through the activation of dendritic cells and M2 macrophage, accompanied by down-regulation of eotaxin and IL-25, and up-regulation of TGF-β and IL-10 in mouse lung epithelial cells. |
| Heo J. et al (9) | ASCs derived exosomes modulated macrophage polarization by upregulating the expression of M2 macrophage markers. |
| Alves V. et al (10) | A single administration of human ASCs induces durable and sustained long-term regulation of inflammatory response in experimental colitis |
| Comella K. et al (11) | First clinical implementation of SVF (fraction derived from lipoaspirate-cell isolation containing ASCs and other endothelial cells) intravenously in a patient with severe psoriasis. The patient did not report any safety concerns and did not experience any severe adverse events. The patient demonstrated a significant decrease in symptoms with a noticeable difference in skin quality appearance. |

**Bibliography**

1. Serejo TRT, Silva-Carvalho AÉ, Braga LD de CF, Neves F de AR, Pereira RW, Carvalho JL de, et al. Assessment of the Immunosuppressive Potential of INF-γ Licensed Adipose Mesenchymal Stem Cells, Their Secretome and Extracellular Vesicles. Cells. 2019 Jan 5;8(1):22.

2. Wang YC, Chen RF, Brandacher G, Lee WPA, Kuo YR. The suppression effect of dendritic cells maturation by adipose-derived stem cells through TGF-β1 related pathway. Exp Cell Res. 2018 Sep 15;370(2):708–17.

3. Gornostaeva AN, Andreeva ER, Bobyleva PI, Buravkova LB. Interaction of allogeneic adipose tissue-derived stromal cells and unstimulated immune cells in vitro: the impact of cell-to-cell contact and hypoxia in the local milieu. Cytotechnology. 2018 Feb 1;70(1):299–312.

4. Zhang W, Feng YL, Pang CY, Lu FA, Wang YF. Transplantation of adipose tissue-derived stem cells ameliorates autoimmune pathogenesis in MRL/lpr mice: Modulation of the balance between Th17 and Treg. Z Rheumatol. 2019 Feb 1;78(1):82–8.

5. Wang Y, Wang X, Zhou X, Zhu Z, Yang J, Liu F. Suppressive effect mediated by human adipose-derived stem cells on T cells involves the activation of JNK. Int J Mol Med. 2019 Jan 1;43(1):177–84.

6. Zhou K, Guo S, Tong S, Sun Q, Li F, Zhang X, et al. Immunosuppression of human adipose-derived stem cells on T cell subsets via the reduction of NF-kappaB activation mediated by PD-L1/PD-1 and Gal-9/TIM-3 Pathways. Stem Cells Dev. 2018 Sep 1;27(17):1191–202.

7. Blazquez R, Sanchez-Margallo FM, de la Rosa O, Dalemans W, Álvarez V, Tarazona R, et al. Immunomodulatory potential of human adipose mesenchymal stem cells derived exosomes on in vitro stimulated T cells. Front Immunol. 2014;5(NOV).

8. Cho KS, Kang SA, Kim SD, Mun SJ, Yu HS, Roh HJ. Dendritic cells and M2 macrophage play an important role in suppression of Th2-mediated inflammation by adipose stem cells-derived extracellular vesicles. Stem Cell Res. 2019 Aug 1;39.

9. Heo JS, Choi Y, Kim HO. Adipose-Derived Mesenchymal Stem Cells Promote M2 Macrophage Phenotype through Exosomes. 2019.

10. Alves VBF, de Sousa BC, Fonseca MTC, Ogata H, Caliári-Oliveira C, Yaochite JNU, et al. A single administration of human adipose tissue-derived mesenchymal stromal cells (MSC) induces durable and sustained long-term regulation of inflammatory response in experimental colitis. Clin Exp Immunol. 2019 May 1;196(2):139–54.

11. Comella K, Parlo M, Daly R, Dominessy K. First-in-man intravenous implantation of stromal vascular fraction in psoriasis: A case study. Int Med Case Rep J. 2018 Mar 21;11:59–64.
